# Supplementary material for: Inference of tissue relative proportions of the breast epithelial cell types luminal progenitor, basal, and luminal mature
Source: Sci Rep. 2021 Dec 8;11:23702. doi: 10.1038/s41598-021-03161-7 (PMC8655091; doi:10.1038/s41598-021-03161-7)
Supplement: Supplementary file 1 — Supplementary Information. [file 41598_2021_3161_MOESM1_ESM.pdf]

Inference of tissue relative proportions of the breast epithelial cell types luminal progenitor, basal, and luminal mature - Supplement

Thomas E. Bartlett, Peiwen Jia, Swati Chandna and Sandipan Roy

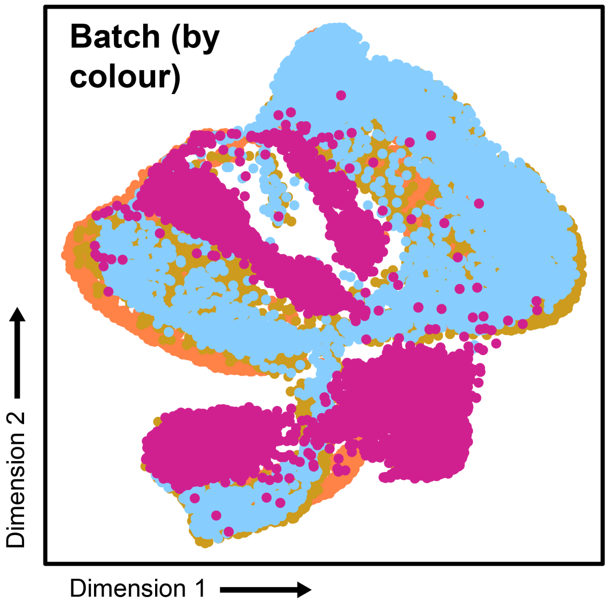

Figure S1: *UMAP projections of the single-cell RNA-seq data from* $n = 13909$ *breast epithelial cells shown in Fig.3, demonstrating batch-effect correction.*

Epithelial subtype	Gene
Basal	*ACTA2*
Basal	*TAGLN*
Basal	*MYL9*
Basal	*TPM2*
Basal	*ACTG2*
Basal	*OXTR*
Basal	*ITGA6*
Basal	*MME*
Basal	*TP63*
Basal	*KRT5*
Basal	*KRT14*
Basal	*KRT17*
Luminal progenitor	*KRT23*
Luminal progenitor	*LTF*
Luminal progenitor	*SLPI*
Luminal progenitor	*RARRES1*
Luminal progenitor	*MMP7*
Luminal progenitor	*ALDH1A3*
Luminal progenitor	*PROM1*
Luminal progenitor	*KRT15*
Luminal progenitor	*PI3*
Luminal progenitor	*S100A9*
Luminal progenitor	*BBOX1*
Luminal progenitor	*GABRP*
Luminal mature	*AREG*
Luminal mature	*AGR2*
Luminal mature	*ANKRD30A*
Luminal mature	*PRLR*
Luminal mature	*PGR*
Luminal mature	*ESR1*
Luminal mature	*FOXA1*
Luminal mature	*PIP*
Luminal mature	*TFF1*
Luminal mature	*TFF3*

Table S1: *Reference genes used for epithelial subtype identification of* $n = 13909$ *breast epithelial cells shown in Fig.3.*
